# Supplementary material for: Fine Mapping and Cloning of a qRA2 Affect the Ratooning Ability in Rice (Oryza sativa L.)
Source: Int J Mol Sci. 2023 Jan 4;24(2):967. doi: 10.3390/ijms24020967 (PMC9863488; doi:10.3390/ijms24020967)
Supplement: Supplementary file 1 [file ijms-24-00967-s001.zip › ijms-2065722-supplementary.pdf]

Supplementary Materials

Table S1. Primer sequences used for synthesizing gRNA spacers and genotyping CRISPR-edited mutants.

Name	Sequence 5′→3′	Purpose
gRNAs-*qRA2*	gtcgctgtgccgctccaccaagg	CRISPR/CAS9
qRA2-F1	CCACAGATCTTCTTCCTTCC	Screening of lines
qRA2-R1	GGTATCCGAGATAGCTGATGG	Screening of lines
*qRA2*cDS-R	ATGGGTTCTCTGGGAGCAG	Gene amplification
*qRA2*cDS-F	CTACGACTCTTTGGCCAGGA	Gene amplification
qRA2-qRT-PCR-F	CCGTCACTACCGCCTCCTCA	RT-qPCR
qRA2-qRT-PCR-R	CGGCGTGTCAAGGTATCCATTC	RT-qPCR
UBIQUITIN-F	AACCAGCTGAGGCCCAAGA	Reference gene
UBIQUITIN-R	ACGATTGATTTAACCAGTCCATGA	Reference gene

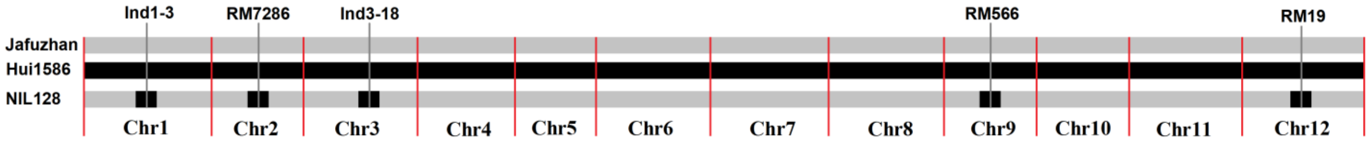

Figure S1. Analysis of the genetic background of NIL128. NIL128 basically recovered the genetic background of the recipient parents except for 5 loci.

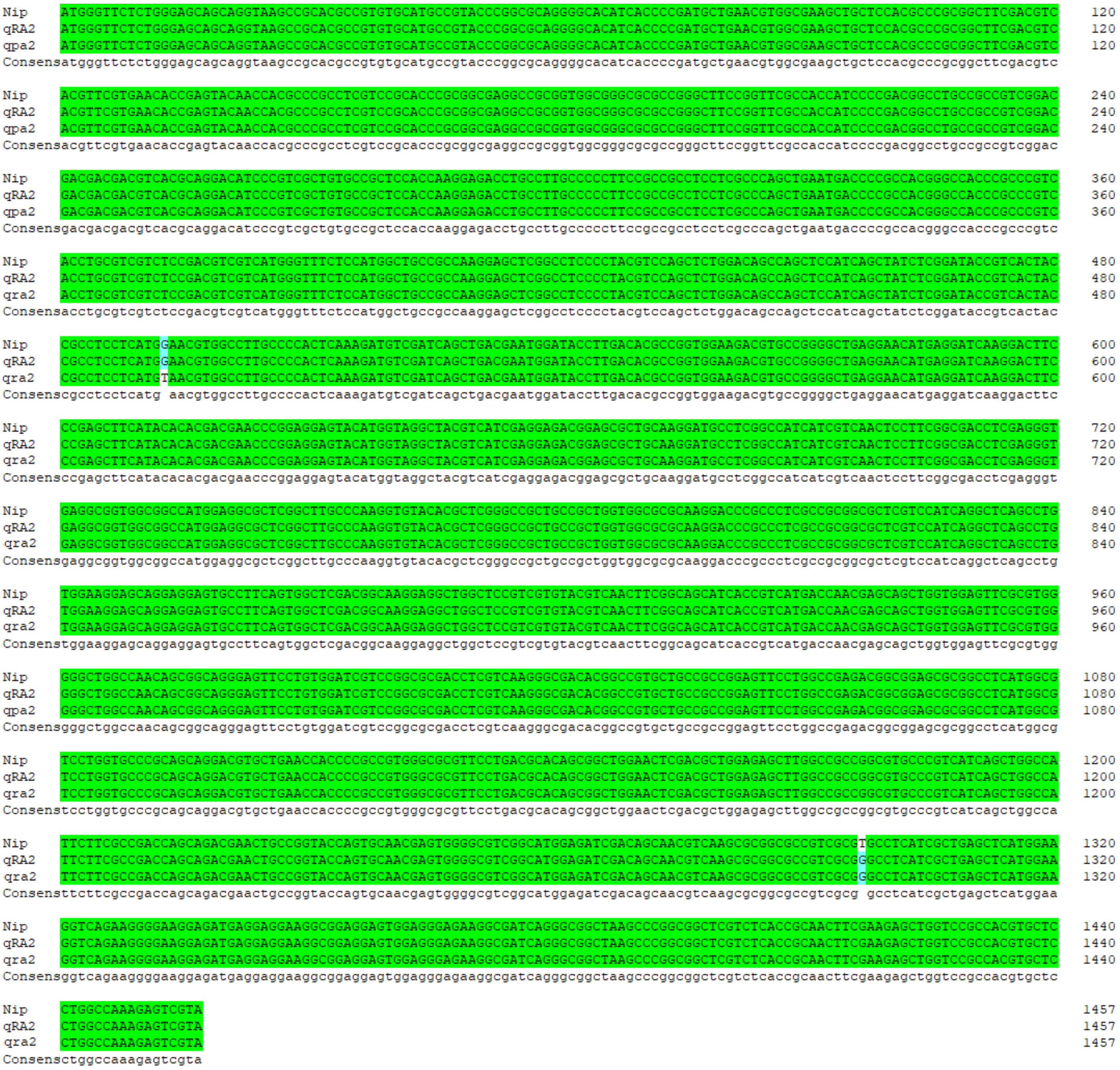

Figure S2. Multiple alignments of nucleic acid sequences. The nucleic acid sequences of Nip, *qRA2* and *qra2* in the LOC_Os02g51930 locus are displayed.

```
Nip     MGSLGAAGKPHAVCMPYPAQGHITPMLNVAKLLHARGFDVTFVNTEYNHARLVRTRGEAAVAGAPGFRFATIPDGLPPSDDDDVTQDIPSLCRSTKETCLAPFRRLLAQLNDPATGHPPV    120
qRA2    MGSLGAAGKPHAVCMPYPAQGHITPMLNVAKLLHARGFDVTFVNTEYNHARLVRTRGEAAVAGAPGFRFATIPDGLPPSDDDDVTQDIPSLCRSTKETCLAPFRRLLAQLNDPATGHPPV    120
qra2    MGSLGAAGKPHAVCMPYPAQGHITPMLNVAKLLHARGFDVTFVNTEYNHARLVRTRGEAAVAGAPGFRFATIPDGLPPSDDDDVTQDIPSLCRSTKETCLAPFRRLLAQLNDPATGHPPV    120
Consensmgslgaagkphavcmpypaqghitpmlnvakllhargfdvtfvnteynharlvrtrgeaavagapgfrfatipdglppsddddvtqdipslcrstketclapfrrllaqlndpatghppv

Nip     TCVVSDVVMGFSMAAAKELGLPYVQLWTASSISYLGYRHYRLLMERGLAPLKDVDQLTNGYLDTPVEDVPGLRNMRIKDFPSFIHTTNPEEYMVGYVIEETERCKDASAIIVNSFGDLEG    240
qRA2    TCVVSDVVMGFSMAAAKELGLPYVQLWTASSISYLGYRHYRLLMERGLAPLKDVDQLTNGYLDTPVEDVPGLRNMRIKDFPSFIHTTNPEEYMVGYVIEETERCKDASAIIVNSFGDLEG    240
qra2    TCVVSDVVMGFSMAAAKELGLPYVQLWTASSISYLGYRHYRLLMR.GLAPLKDVDQLTNGYLDTPVEDVPGLRNMRIKDFPSFIHTTNPEEYMVGYVIEETERCKDASAIIVNSFGDLEG    239
Consenstcvvsdvvmgfsmaaakelglpyvqlwtassisylgyrhyrllm  glaplkdvdqltngyldtpvedvpglrnmrikdfpsfihttnpeeymvgyvieeterckdasaiivnsfgdleg

Nip     EAVAAMEALGLPKVYTLGPLPLVARKDPPSPRRSSIRLSLWKEQEECLQWLDGKEAGSVVYVNFGSITVMTNEQLVEFAWGLANSGREFLWIVRRDLVKGDTAVLPPEFLAETAERGLMA    360
qRA2    EAVAAMEALGLPKVYTLGPLPLVARKDPPSPRRSSIRLSLWKEQEECLQWLDGKEAGSVVYVNFGSITVMTNEQLVEFAWGLANSGREFLWIVRRDLVKGDTAVLPPEFLAETAERGLMA    360
qra2    EAVAAMEALGLPKVYTLGPLPLVARKDPPSPRRSSIRLSLWKEQEECLQWLDGKEAGSVVYVNFGSITVMTNEQLVEFAWGLANSGREFLWIVRRDLVKGDTAVLPPEFLAETAERGLMA    359
Consenseavaamealglpkvytlgplplvarkdppsprrssirlslwkeqeeclqwldgkeagsvvyvnfgsitvmtneqlvefawglansgreflwivrrdlvkgdtavlppeflaetaerglma

Nip     SWCPQQDVLNHPAVGAFLTHSGWNSTLESLAAGVPVISWPFFADQQTNCRYQCNEWGVGMEIDSNVKRGAVACLIAELMEGQKGKEMRRKAEEWREKAIRAAKPGGSSHRNFEELVRHVL    480
qRA2    SWCPQQDVLNHPAVGAFLTHSGWNSTLESLAAGVPVISWPFFADQQTNCRYQCNEWGVGMEIDSNVKRGAVAGLIAELMEGQKGKEMRRKAEEWREKAIRAAKPGGSSHRNFEELVRHVL    480
qra2    SWCPQQDVLNHPAVGAFLTHSGWNSTLESLAAGVPVISWPFFADQQTNCRYQCNEWGVGMEIDSNVKRGAVAGLIAELMEGQKGKEMRRKAEEWREKAIRAAKPGGSSHRNFEELVRHVL    479
Consensswcpqqdvlnhpavgaflthsgwnstleslaagvpviswpffadqqtncryqcnewgvgmeidsnvkrgava liaelmegqkgkemrrkaeewrekairaakpggsshrnfeelvrhvl

Nip     LAKE                                                                                                                        484
qRA2    LAKE                                                                                                                        484
qra2    LAKE                                                                                                                        483
Consenslake
```

Figure S3. Multiple alignments of amino acid sequences. Amino acid sequences of Nip, *qRA2* and *qra2* in the LOC_Os02g51930 locus are displayed.
